# IL-7 plays a critical role for the homeostasis of allergen-specific memory CD4 T cells in the lung and airways

**DOI:** 10.1038/s41598-017-11492-7

**Published:** 2017-09-11

**Authors:** Seung-min Yeon, Lea Halim, Anmol Chandele, Curtis J. Perry, Sang Hoon Kim, Sun-Uk Kim, Youngjoo Byun, Soon Hong Yuk, Susan M. Kaech, Yong Woo Jung

**Affiliations:** 1 Department of Pharmacy, Korea University, Sejong-si, Korea; 2 Department of Immunobiology, Yale University School of Medicine, New Haven, CT USA; 3 Howard Hughes Medical Institute, Yale University School of Medicine, New Haven, CT USA; 4 ICGEB-Emory Vaccine Center, International Center for Genetic Engineering and Biotechnology, Aruna Asaf Ali Marg, New Delhi, 110067 India; 5 National Primate Research Center and Futuristic Animal Resource & Research Center, Korea Research Institute of Bioscience and Biotechnology, Cheongju-si, Chungcheongbuk-do Republic of Korea

## Abstract

Memory T cells respond rapidly to repeated antigen exposure and can maintain their population for extended periods through self-renewal. These characteristics of memory T cells have mainly been studied during viral infections, whereas their existence and functions in allergic diseases have been studied incompletely. Since allergic patients can suffer repeated relapses caused by intermittent allergen exposure, we hypothesized that allergen- specific memory Th2 cells are present and the factors necessary for the maintenance of these cells are provided by the lung and airways. Using a murine model of airway inflammation, we found that allergen-specific CD4 T cells survived longer than 70 days in the lung and airways in an IL-7 dependent fashion. These T cells showing homeostatic proliferation were largely found in the mediastinal lymph node (mLN), rather than the airways; however, cells residing in the lung and airways developed recall responses successfully. We also found that CD4 T cells exhibited differential phenotypes in the mLN and in the lung. Altogether, we believe that allergen-specific memory T cells reside and function in the lung and airways, while their numbers are replenished through homeostatic turnover in the mLNs. Furthermore, we determined that IL-7 signaling is important for the homeostasis of these cells.

## Introduction

Allergic asthma is a chronic allergic disease characterized by eosinophilic airway inflammation and hyperreactivity^1–4^. Type 2 helper T (Th2) cells have been identified as orchestrating this pathogenesis by providing cytokines such as IL-4, IL-5, and IL-13^5, 6^. IL-4 stimulates B cells to switch the isotype to IgE. IL-5 induces the development and recruitment of eosinophils into the airway. IL-13 induces airway hyperreactivity and mucus production by epithelial cells. Therefore, studies of Th2 cells have focused on their development and function in allergic responses. However, the survival of Th2 cells in the lung and airway after the allergic responses has been incompletely defined.

T cells with longevity have been found for the first time in the form of memory CD8 T cells, which have been well characterized during viral infections^7–11^. These cells live long because they have extended half-lives and turn over homeostatically. To achieve longevity, memory T cells rely on IL-7 and IL-15 signalings^12–18^. IL-7 is required for the development and survival of memory CD8 T cells. Additionally, IL-7 in conjunction with IL-15 is crucial for the homeostatic turnover of these cells. On the other hand, virus-specific CD4 memory T cells are considered to have shorter half-lives compared to CD8 T cells. During an influenza infection, CD4 T cells greatly expanded in the lung and airways, but did not survive more than a month^19^. Therefore, the survival of memory T cells and its underlying mechanisms may vary depending on the type of memory T cells.

Memory T cells can also be categorized based on their roles during secondary responses. While central memory T cells (TCM) circulate among secondary lymphoid organs, effector memory T cells (TEM) search for their cognate antigen in the non-lymphoid organs^20, 21^. These two subsets also show differential phenotypes. TCMs express high levels of CCR7 and CD62L, while TEMs are CCR7^lo^ CD62L^lo^. Recently, resident memory T cells (TRM) have been identified as residing in non-lymphoid organs such as the skin, lung, brain, and salivary gland^22–29^. These TRM cells do not express lymphoid homing molecules, and were identified as CD69^hi^ CD103^hi^ in most of the organs where they were detected^22–26, 30, 31^. These cells have been highlighted for their role in local protection against secondary infections by responding robustly as a first line of defense.

Although memory T cells have been intensively characterized in response to infections, the importance of these cells in allergic diseases remains to be elucidated. Herein, we investigated the characteristics of Th2 memory cells using a murine model of airway inflammation. We observed that the allergen-specific CD4 T cells could survive longer than 70 days in the lung and airway. We also found that the maintenance of their homeostasis was dependent on IL-7 signaling. Lastly, the allergen-specific memory T cells in the lung and airways were sufficient to induce eosinophilic inflammation.

## Results

### Allergen-specific memory CD4 T cells are present in the lung and airway

**Figure 1:**
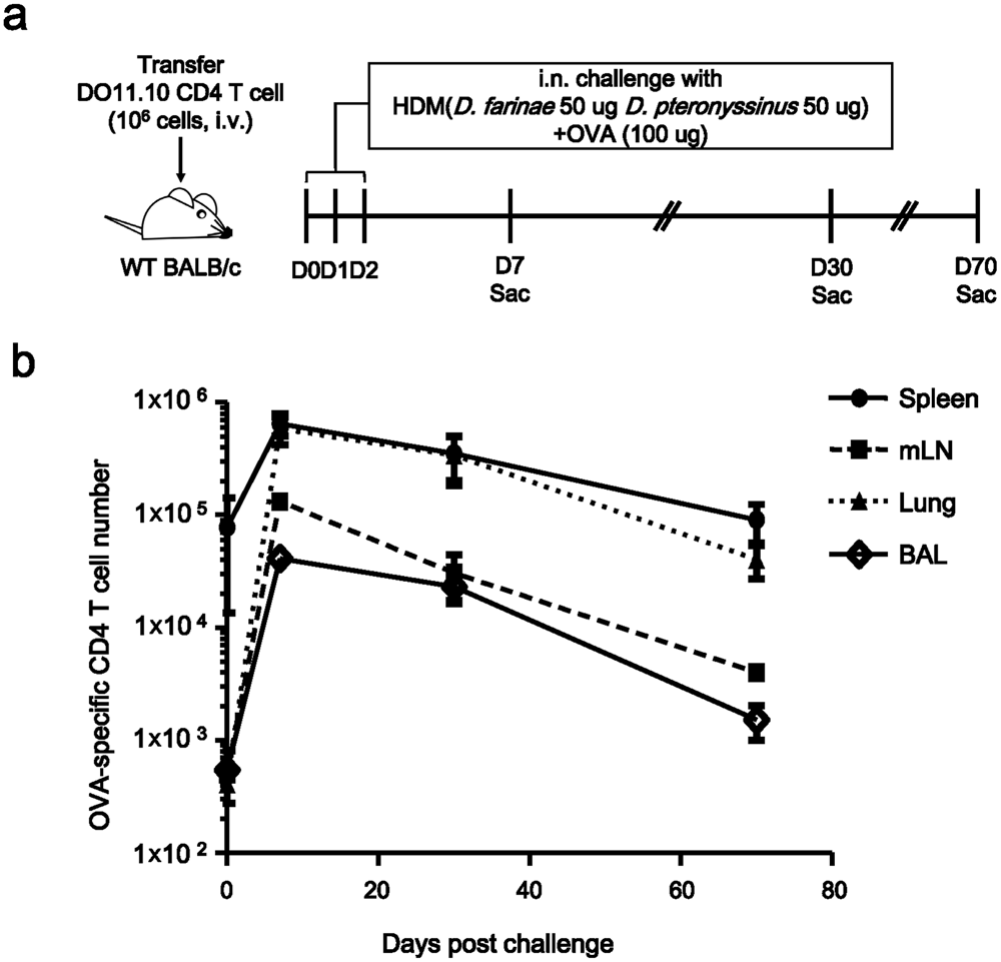
Allergen-specific memory CD4 T cells survived more than 70 days in the lung and airways. (**a**) Shown is the immunization and analysis timeline. (**b**) Lymphocytes were isolated from the organs as indicated and cells were stained with anti-CD4 and anti-TCR DO11.10 antibody. The numbers of OVA-specific CD4 T cells were calculated using flow cytometry. These data represent Mean ± SEM. N = 3, 14, 15, and 9 mice (represent day 0, 7, 30 and 70 each) for each group and we presented pooled data of 9 independent experiments.

In order to test our hypothesis that allergen-specific memory CD4 T cells are present in the lung and airways, we treated mice containing OVA-specific CD4 T cells with a mixture of house dust mite (HDM) and ovalbumin (OVA) by the intranasal (i.n.) route for 3 days (Fig. 1a). We chose to challenge mice with a mixture of HDM and OVA because HDM has been shown to induce eosinophilic airway inflammation in both humans and mice^32–36^. In addition, the treatment with OVA as an allergen enabled us to trace OVA-specific DO11.10 cells by using anti-clonotypic antibody KJ1-26. On days 7, 30, and 70 post challenge these mice were sacrificed to measure the number of OVA-specific CD4 T cells in the spleen, mediastinal lymph node (mLN), lung, and bronchoalveolar lavage (BAL) fluid. We found that the number of these cells peaked on day 7 and declined on day 30 and 70 in these organs (Fig. 1b). We also examined if these long-lived allergen-specific CD4 T cells can induce eosinophilic inflammation following rechallenge. Mice sensitized for 3 days from day 0 were rechallenged with a mixture of HDM and OVA for 2 days from day 22, and sacrificed on day 25 (Supplementary Fig. 1a). We observed that the number of OVA-specific CD4 T cells increased in response to recall, and the mice developed eosinophilic inflammation (Supplementary Fig. 1b and c). These data suggest that the allergen-specific CD4 T cells survive for an extended period, with a recall ability, in the lung and airways.

### The proliferation of allergen-specific CD4 T cells undergoing memory CD4 T cell maturation occurs primarily in the mLN

**Figure 2:**
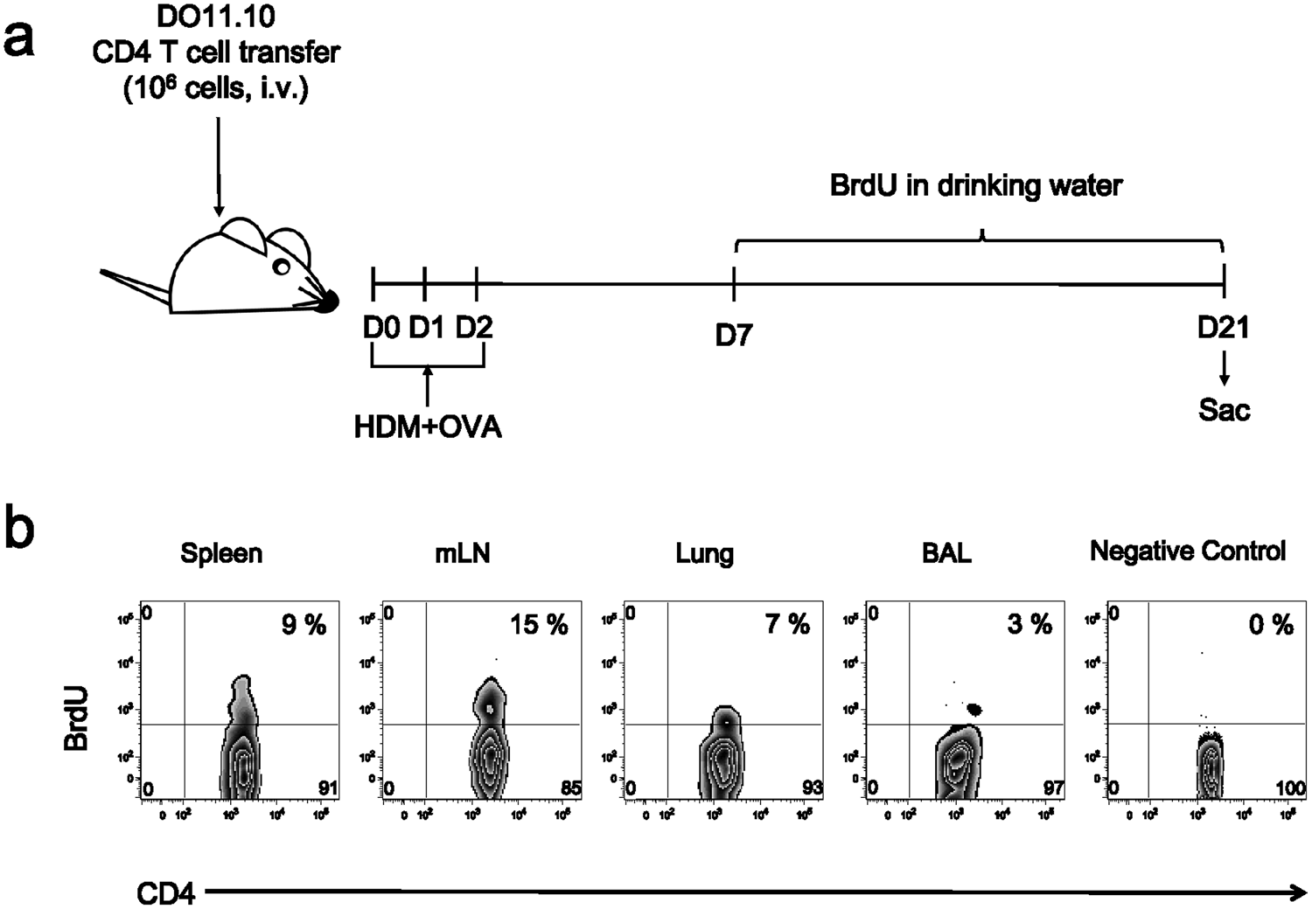
The proliferation of allergen-specific CD4 T cells occurred primarily in the mLN during the maturation of memory CD4 T cells. (**a**) From day 7 post challenge, mice were given BrdU for 14 days. On day 22, mice were sacrificed and OVA-specific cells were analyzed using flow cytometry. (**b**) Representative contour plots of BrdU+ cells from OVA-specific CD4 T cells of spleen, mLN, lung, and airways. Similar experiments were performed twice.

Since we determined the longevity of allergen-specific CD4 T cells in the lung and airways, we investigated if these cells undergo homeostatic turnover to maintain their numbers^37–39^. We fed mice with bromodeoxyuridine (BrdU) in their drinking water between days 7 and 21 post challenge, and then sacrificed them on day 21. The numbers of BrdU+ cells in the spleen, mLN, lung, and airways were measured by flow cytometry (Fig. 2a). Although BrdU+ OVA-specific CD4 T cells were found in every organ we analyzed, the ratio of these cells in the mLN was approximately 15%. In agreement with a previous report showing that T cells in the airways do not proliferate^40^, the lung and airways contain only 7% and 3% of these cells, respectively (Fig. 2b). Altogether, we suggest that allergen-specific CD4 T cells in the process of memory T cell maturation proliferate mainly in the mLN, while these cells are continuously recruited into the lung and airways to maintain their numbers in these organs.

### Allergen-specific CD4 T cells showed differential phenotypes depending on their environments

**Figure 3:**
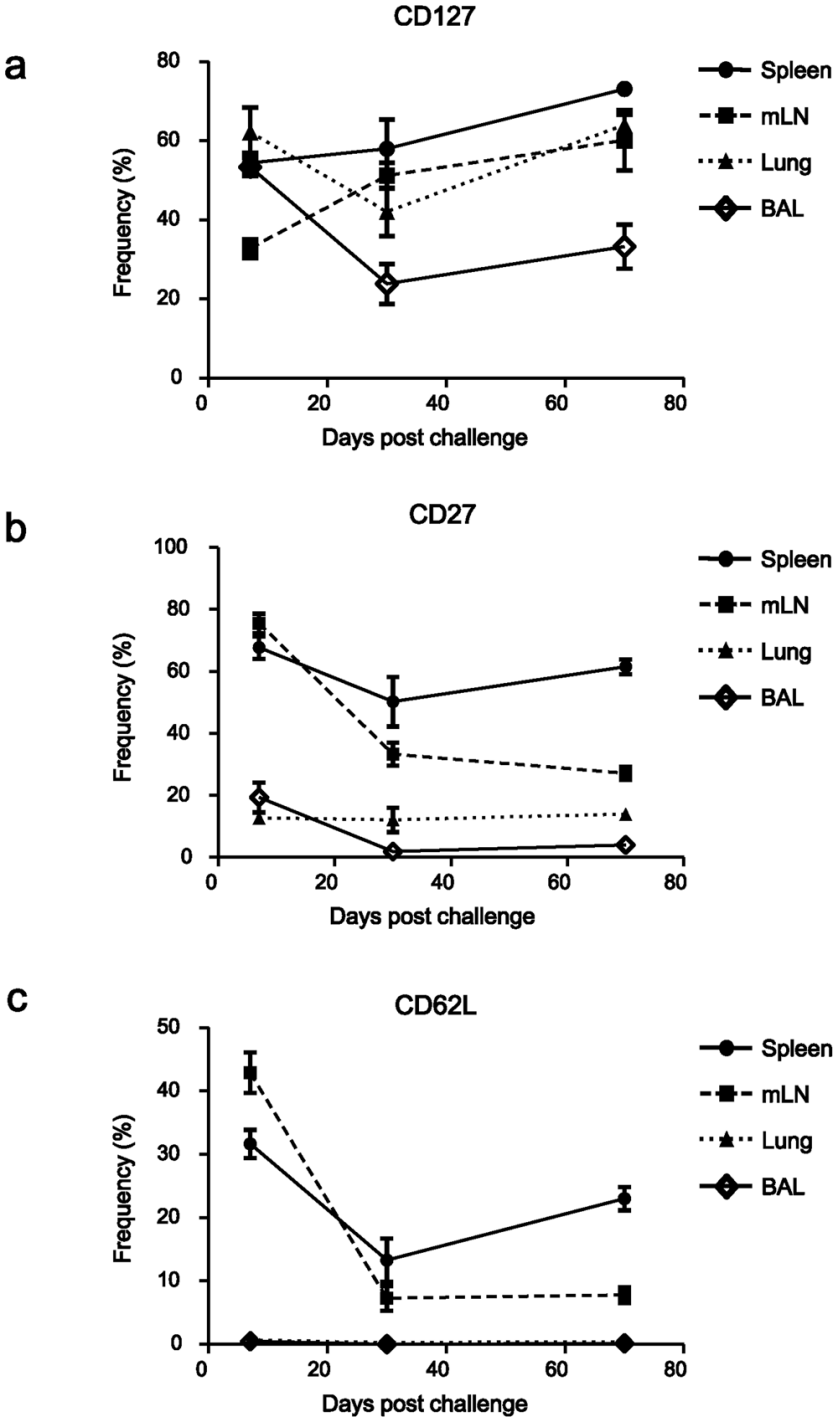
Allergen-specific memory CD4 T cells in the lung and airways showed unique phenotypes. The expressions of (**a**) CD127, (**b**) CD27, and (**c**) CD62L of OVA-specific memory CD4 T cell were examined from the spleen, mLN, lung, and BAL on days 7, 30, and 70 post challenge using flow cytometry. These data represent Mean ± SEM. Four independent experimental data using at least 8 mice per each time point were compiled.

Since the allergen-specific CD4 T cells in the lung and airways showed recall ability and homeostatic turnover, we further characterized the phenotypes of OVA-specific CD4 T cells using flow cytometry. On days 7, 30, and 70 after challenge with a mixture of HDM and OVA, mice were sacrificed and CD4 T cells were then stained with surface markers that are classically used to study memory CD8 T cell phenotype, including CD127, CD27, CD62L, CD122, and KLRG1. No significant differences were found in the expression of CD122 and KLRG1 during the course of experiment. We observed that a relatively constant ratio of CD127^hi^ cells was maintained in the spleen, mLN (25~45%), and lung (50~70%) during the course of airway inflammation. On the other hand, in the airways there was a dramatic decline of CD127^hi^ OVA-specific CD4 T cells (50%→15%) and then 15% were maintained during the memory phase (Fig. 3a). While the CD27^hi^ cell ratio was maintained in the spleen during the course of the experiment (>60%), lung (7~17%), and airways (>10%), the mLN showed a dramatic decline in the CD27^hi^ ratio (70%→25%) (Fig. 3b). Lastly, we observed that the CD62L^hi^ cell ratio was maintained in the spleen (20~40%), while a dramatic decline of the CD62L^hi^ ratio, which is unusual, was observed in the mLN (60%→7%), and fewer than 1% of these cells were CD62L^hi^ in the lung and airways (Fig. 3c and Supplementary Fig. 2). We also found that the OVA-specific CD4 T cells showed different phenotypes when they were challenged with either OVA only or HDM only (data not shown). In summary, the allergen-specific CD4 T cells in the mLN, lung, and airways showed differential phenotypes depending on their environments.

### Allergen-specific CD4 T cells require IL-7 signaling for their homeostasis

**Figure 4:**
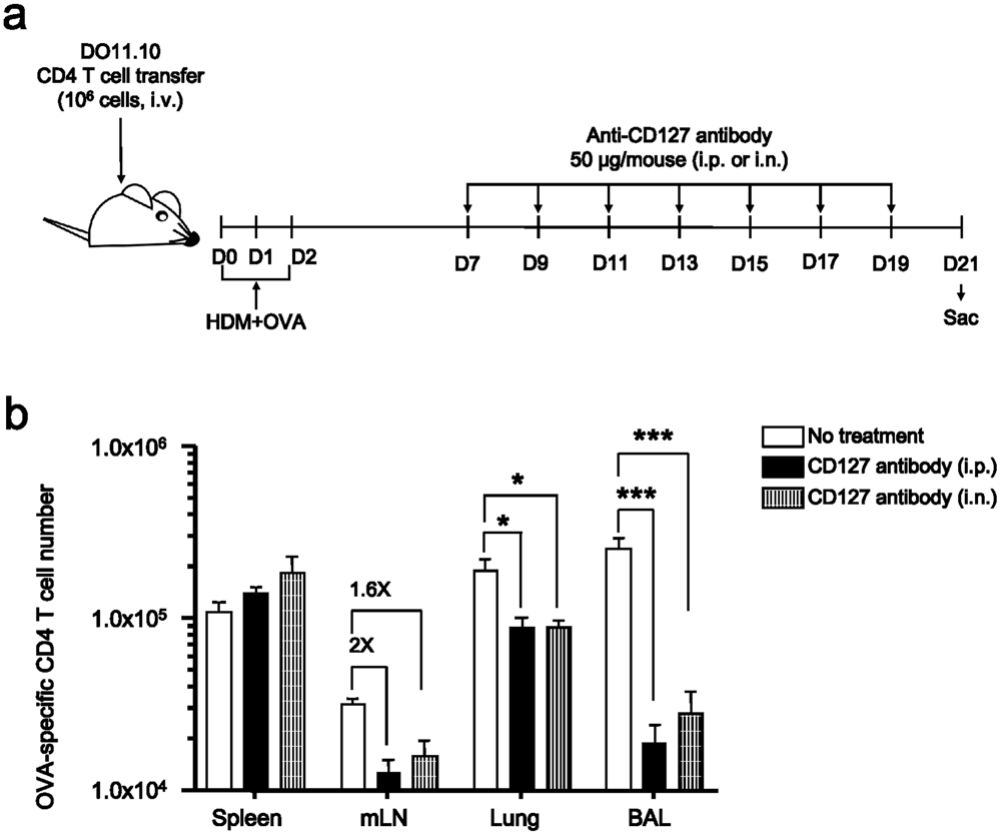
The number of allergen-specific memory CD4 T cells decreased after the inhibition of CD127 signaling. (**a**) Allergen challenged mice were treated with an anti-CD127 antibody either i.p. or i.n. every other day from day 7 to day 19. (**b**) The numbers of OVA-specific CD4 T cells were determined using FACS analysis. We compiled three experimental data sets with 9 mice in individual groups. Bar graph depicts Mean ± SEM. (*P < 0.05; ***P < 0.001).

After we determined the longevity of allergen-specific Th2 cells, we next examined whether IL-7 is importantly involved in the homeostasis of these cells. We chose this cytokine based on its roles in the development and maintenance of memory CD8 T cells. In addition, the expression of CD127, IL-7 receptor α, was observed on OVA-specific CD4 T cells from D30 post challenge, and these cells showed the increased levels of phosphorylated STAT5 signaling when stimulated with IL-7 *in vitro* (Supplementary Fig. 3). In order to inhibit IL-7 signaling *in vivo*, we injected CD127 blocking monoclonal antibody (mAb, clone A7R34) into mice via either the i.n. or intraperitoneal (i.p.) route, once every two days for two weeks beginning from day 7 post allergen challenge (Fig. 4a). A group of OVA-challenged mice received PBS as a negative control. Mice were sacrificed on day 27 post challenge and the number of OVA-specific CD4 T cells was compared. The number of OVA-specific CD4 T cells in the mLN showed a 2-fold decrease in the i.p. group and a 1.6-fold decrease in the i.n. group compared to the control. The OVA-specific CD4 T cells in the lung decreased 2-fold compared to the control in both the i.p and i.n. groups. In the airways, the number of OVA-specific CD4 T cells decreased 10-fold in the i.p. group and 6-fold in the i.n. group compared to the control (Fig. 4b). These data suggest that IL-7 signaling plays a crucial role in the homeostasis of allergen-specific CD4 T cells in the mLN, lung, and airways.

### Allergen-specific memory CD4 T cells that reside in the lung can develop eosinophilic airway inflammation

**Figure 5:**
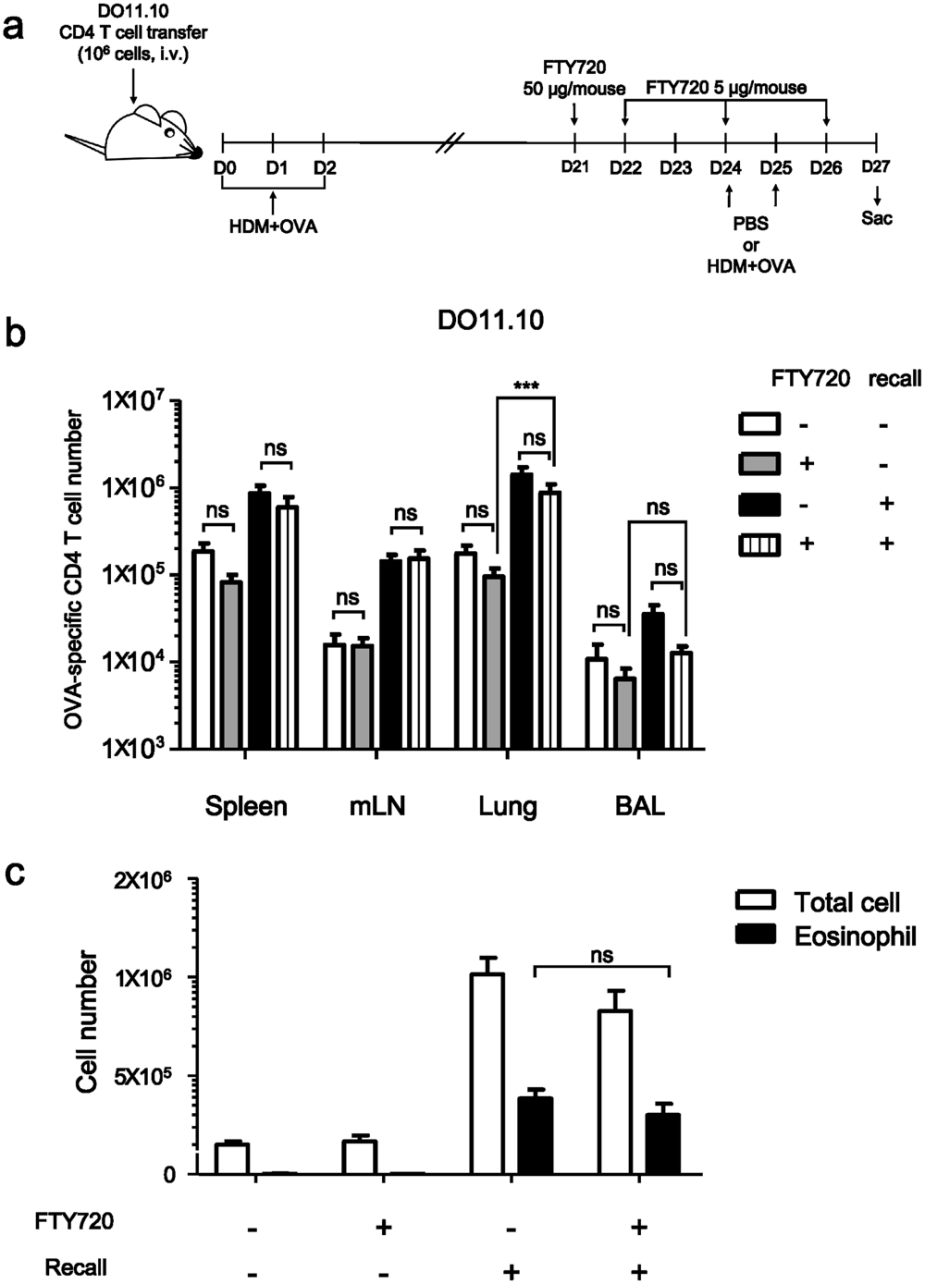
Allergen-specific memory CD4 T cells residing in the lung and airways induced eosinophilic inflammation. (**a**) Mice were challenged and treated with FTY720 on days 21, 22, 24, and 26 post challenge. Mice were rechallenged with PBS or a mixture of HDM and OVA on day 24 and 25, and then sacrificed on day 27. (**b**) The number of OVA-specific CD4 T cells was counted. Bar graph depicts Mean ± SEM. (**c**) Total cell numbers were counted using trypan blue staining. Eosinophil numbers were counted by cytospin and Diff-Quick staining. Bar graph depicts Mean ± SEM (***P < 0.001; ns: not significant). Similar experiments were repeated three times. Eight mice for each group were used and three experimental data sets were compiled.

We further investigated whether the allergen-specific CD4 T cells that reside in the lung and airways are sufficient to develop eosinophilic inflammation in response to recall challenge. In order to inhibit the recruitment of T cells into the lung, we treated these mice with FTY720 on days 21, 22, 24, and 26 post challenge, because this chemical blocks lymphocyte egress from lymph node to tissue. During this period, we bled mice twice and confirmed that the number of circulating OVA-specific CD4 T cells is limited. These mice were rechallenged on days 24 and 25 and then sacrificed on day 27 (Fig. 5a). In every organ we examined, the number of OVA-specific CD4 T cells was not changed by treatment with FTY720. Further, the mice treated with FTY720 and rechallenged showed significantly higher OVA-specific CD4 T cell numbers compared to the mice given FTY720 without rechallenge (Fig. 5b); this group of mice also showed normal eosinophilic inflammation in the airways (Fig. 5c). These data suggest that the allergen-specific memory CD4 T cells that reside in the lung and airways can develop eosinophilic inflammation upon rechallenge.

## Discussion

This study investigated the characteristics of allergen-specific memory CD4 T cells in the lung and airways using a murine model of airway inflammation. We found that allergen-specific memory CD4 T cells survive longer than 70 days in the lung and airways. These cells required IL-7 signaling to maintain their homeostasis, especially in the lung and airways. We also found that the allergen-specific memory CD4 T cells in the lung induce eosinophilic inflammation.

The presence of memory Th2 cells in a mouse model of allergic asthma was initially suggested based on the observation that Th2 memory responses were found in the spleen and lung. Mojtabavi’s group first identified Th2 memory responses using asthmatic mice that showed eosinophilic inflammation airway hyperreactivity and IgE production 400 days after the initial challenge with aerosolized allergen^41^. Another report suggested the presence of memory Th2 cells that are CD62L^lo^ CXCR3^lo^ and produce IL-5 to induce eosinophilic inflammation^42^. In allergic asthma mice, which were sensitized with a mixture of OVA and alum i.p., the memory Th2 cells were also suggested to exist and able to induce a recall response by showing eosinophilia in the airways 60 days after the last challenge^43^. In agreement with previous data, our study showed that allergen-specific CD4 T cells survived more than 70 days in the lung and airways. Although previous studies provided important evidence for the presence of memory Th2 cells following *in vitro* stimulation of CD4 T cells, we employed an i.n. allergen challenge to see if the allergen-specific memory T cells could be demonstrated *in vivo*. Furthermore, the allergen-specific memory CD4 T cells in our data showed different phenotypes depending on their environments such as the spleen, LN, lung, and airways.

It has been suggested that memory CD4 T cells found in the lung and airways are short-lived in response to viral infections, whereas CD8 T cells survive longer^19^. Contrary to this previous report, we found that allergen-specific CD4 T cells survive more than 70 days in the lung and airways. Although it is not clear how memory CD4 T cells in asthmatic lungs can be maintained for an extended period, it is plausible that the homeostatic proliferation of T cells in the LNs and migration into the lung and airways leads to the long-term maintenance of memory T cells in the lung and airways. This suggestion is based on the previous finding that these T cells are recruited during the steady state in order to supplement the memory T cells that are cleared in the lung and airways after an acute viral infection^44^. We also made a similar observation that the homeostatic proliferation of allergen-specific CD4 T cells occurs primarily in the mLN, but not in the airways. This may indicate that the allergen-specific memory T cells in the LN are continuously recruited to the lung and airways to maintain the number of these cells if they undergo cell death or excretion from the airways.

We further investigated which factors may contribute to the long-term maintenance of memory T cells. It has been suggested that IL-7 and IL-15 are critical factors driving cell survival and homeostatic proliferation of memory T cells in the secondary lymphoid organs^12–18^. In the case of the lung and airways, we observed that long-term maintenance of allergen-specific CD4 T cells requires IL-7 signaling suggesting that this cytokine can support the different types of memory T cells in non-lymphoid organs.

Recent studies have addressed the crucial roles of memory CD8 T cells residing in non-lymphoid organs such as the skin, vaginal mucosa, gut, brain, and lung in inducing secondary responses to infections in these organs^22–29, 45^. Lung-resident CD4 T cells with CD69^+^ CD11a^hi^ have also been found, independent of T cells from circulating and secondary lymphoid organs against viral infection^46^. Although we did not see CD69 and CD11a expression in memory CD4 T cells in the allergen challenged lung, we observed that these cells were sufficient to develop airway inflammation without cell recruitment from the circulation. This suggests that the allergen-specific CD4 TRM cells can develop in the lung.

**Figure 6:**
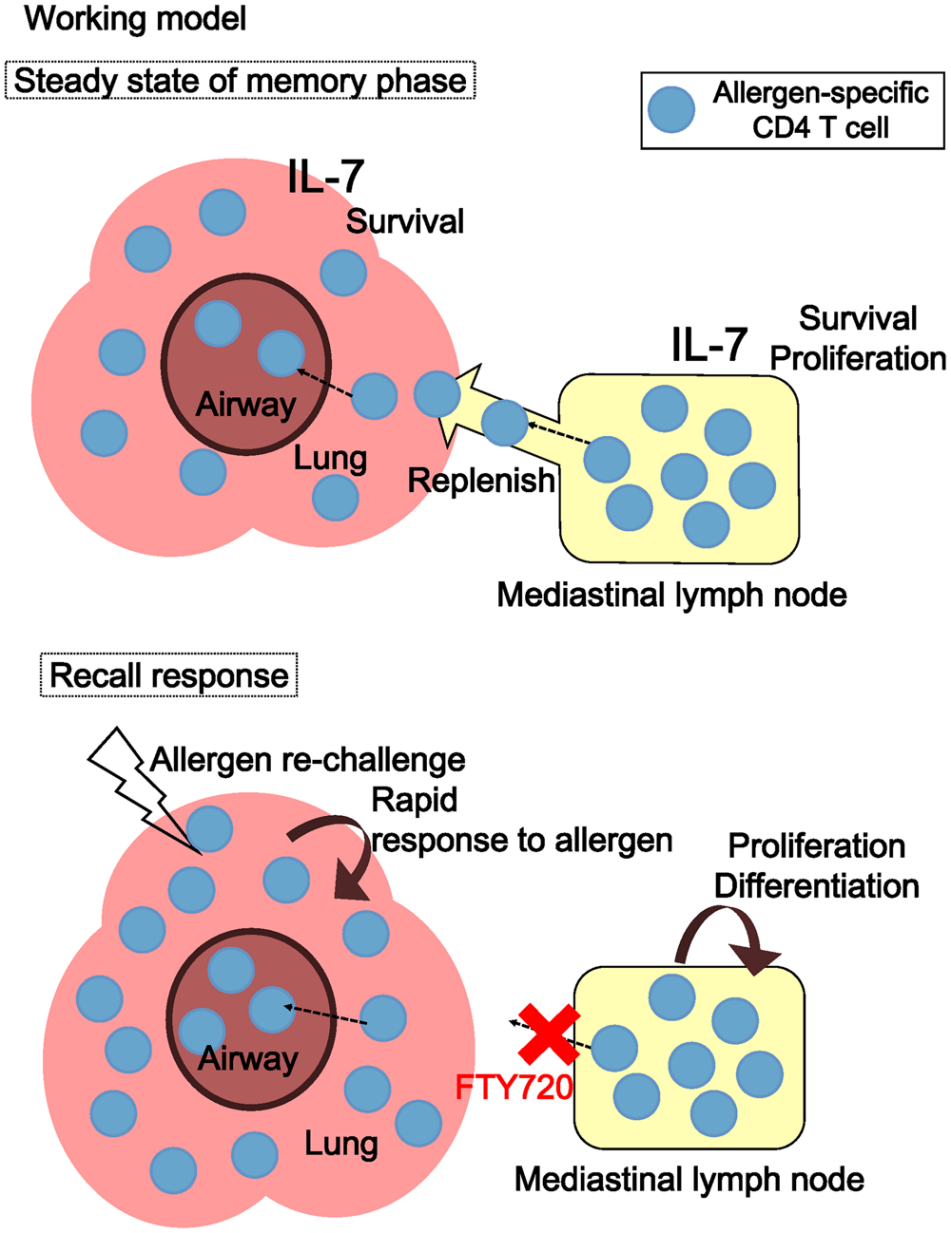
Working model. The allergen-specific memory Th2 cells are developed in the mLN, lung, and airways. These allergen-specific memory Th2 cells survive in the mLN, lung, and airways depending on IL-7 signaling. When allergen recall occurs, the allergen-specific memory Th2 cells that reside in the lung and airways respond rapidly. Then, the allergen-specific Th2 cells are recruited from the mLN to the lung and airways in order to replenish memory Th2 cells cleared from the lung and airways. This model describes how allergen-specific memory Th2 cells keep their long-term maintenance in the lung and airways.

Based on our observations, we describe a model for allergen-specific memory Th2 cell survival in the lung and mLN, shown in Fig. 6. In steady state, the developed allergen-specific memory Th2 cells survive and proliferate in the mLN in response to IL-7 signaling. On the other hand, the allergen-specific memory Th2 cells in the lung are replenished by cell recruitment from mLN whenever these cells are cleared from the lung, such as through cell excretion to the airways. However, the lung resident cells can respond rapidly to allergen recall without cell recruitment from the mLN.

In summary, our research characterized the memory Th2 cells in allergic diseases and suggested the presence of lung-resident memory Th2 cells that depend on IL-7 signalings. While the phenotypes and the other factors required for the survival of these cells in the lung still remain to be investigated, this offers a better understanding of chronic allergic diseases and suggests the possibility of novel drug targets for treatment.

## Materials and Methods

### Mice and Materials

DO11.10 mice were obtained from KAIST. Five-week-old female BALB/c mice were purchased from Orientbio. These mice were maintained and bred under specific pathogen free conditions. The Institutional Animal Care and Use Committees approved all animal protocols at the Korea University and Yale University. We carried out all experiments in accordance with the approved guidelines. Mixtures of HDM extract (*Dermatophagoides farinae* and *Dermatophagoides pteronyssinus*, GREER®) were suspended in distilled water. OVA (Sigma Aldrich) was suspended with phosphate buffered saline and filtered with 0.20-μm pore syringe filter (Satorius stedim biotech). These materials were stored at −20 °C until use.

### Airway inflammation model

DO11.10 mice were sacrificed and splenocytes were isolated. 1 × 10^6^ DO11.10 cells were transferred to naïve BALB/c mice i.v. The day after the transfer, mice were challenged for 3 days with a mixture of HDM (50 μg of *Dermatophagoides farinae* and *Dermatophagoides pteronyssinus* each) and 100 μg of OVA (grade V) i.n. The same mixture was given intranasally for 2 days in recall response. BAL was conducted to isolate lymphocytes from the airways of allergic asthma mice. Isolated lymphocytes from the airways were prepared on the slide glass by cytospin and then stained by Diff-quik staining (Sysmax). In order to inhibit CD127 signaling, 50 μg of CD127 mAb (clone A7R34) was injected via either i.p. or i.n. route as indicated. For FTY720 experiments, mice were treated with FTY720 (Sigma Aldrich) on indicated days i.p.

### Flow-cytometry analysis

BrdU (Sigma Aldrich) was added at 1 mg/mL in the drinking water on day 7 after allergen challenge and BrdU incorporation was examined according to the manufacturer’s instructions (BD). Single cell suspensions were prepared from the lung, mLN, and spleen as described previously^47^. These cells were counted using a hemocytometer and stained followed by flow cytometry using LSRFortessa^TM^ (BD). Following antibodies with fluorescent dye were used: anti-CD4 (PE, GK1.5, BioLegend), anti-CD4 (FITC, RM4-5, BioLegend), anti-TCR DO11.10 (APC, KJ1-26, eBioscience), anti-CD27 (Pacific Blue, BioLegend), anti-CD127 (PE-Cy7, BioLegend), anti-CD62L (APC-Cy7, BioLegend). The plots were analyzed with FlowJo (Tree Star Inc., Ver. 9.7.6).

## Electronic supplementary material

Supplementary Information
