# Supplementary material for: IL-7 plays a critical role for the homeostasis of allergen-specific memory CD4 T cells in the lung and airways
Source: Sci Rep. 2017 Sep 11;7:11155. doi: 10.1038/s41598-017-11492-7 (PMC5593957; doi:10.1038/s41598-017-11492-7)
Supplement: Supplementary file 1 — Supplementary Information [file 41598_2017_11492_MOESM1_ESM.pdf]

IL-7 plays a critical role for the homeostasis of allergen-specific memory CD4 T cells in the lung and airways

Seung-min Yeon[1,*], Lea Halim[2,*], Anmol Chandele[2,4], Curtis J. Perry[2], Sang Hoon Kim[1], Sun-Uk Kim[5], Youngjoo Byun[1], Soon Hong Yuk[1], Susan M. Kaech[2,3], Yong Woo Jung[1]

[1]Department of Pharmacy, Korea University, Sejong-si, Korea

[2]Department of Immunobiology, [3]Howard Hughes Medical Institute, Yale University School of Medicine, CT, USA

[4] ICGEB-Emory Vaccine Center, International Center for Genetic Engineering and Biotechnology, Aruna Asaf Ali Marg, New Delhi – 110067, India

[5] National Primate Research Center & Futuristic Animal Resource & Research Center, Korea Research Institute of Bioscience and Biotechnology, Republic of Korea

*** These authors contributed equally.**

Address correspondence to:

Yong Woo Jung, yjung@korea.ac.kr

Supplementary Fig. 1

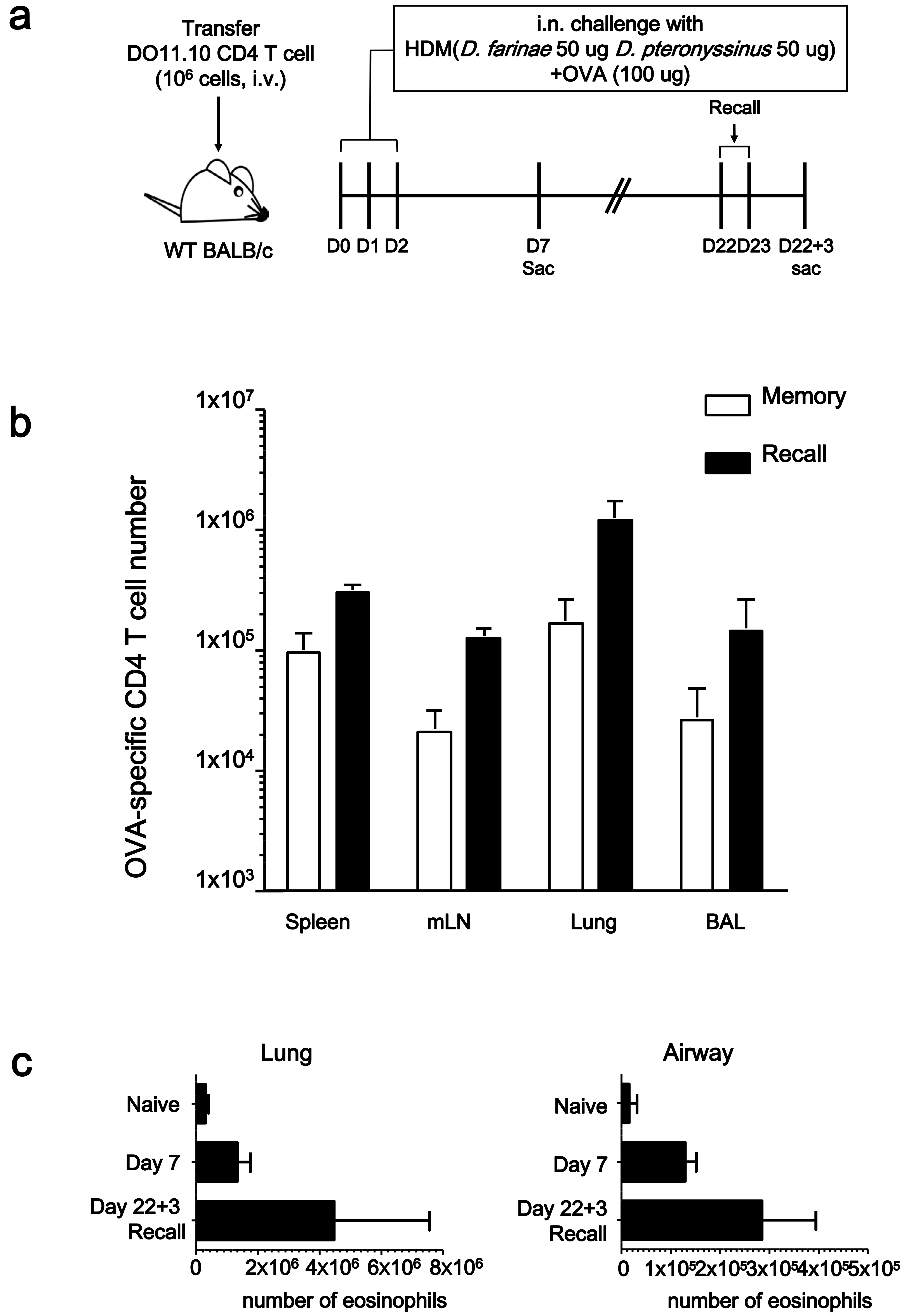

Supplementary Fig. 2

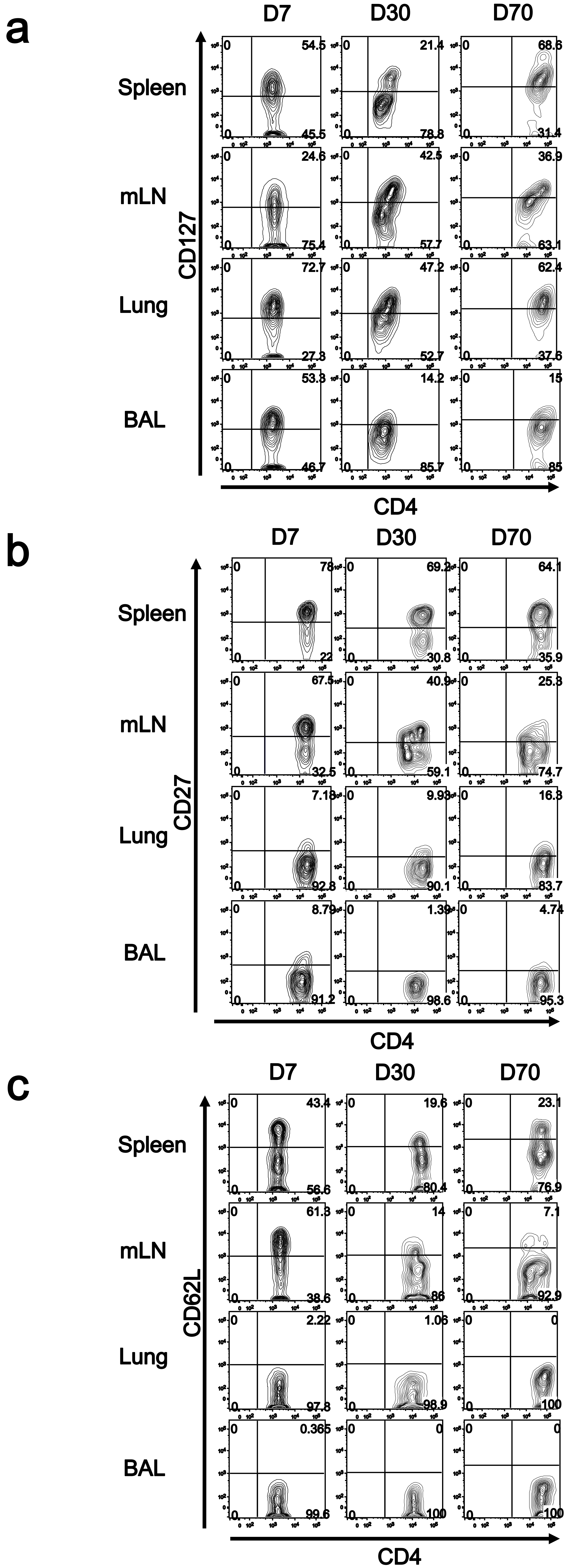

Supplementary Fig. 3

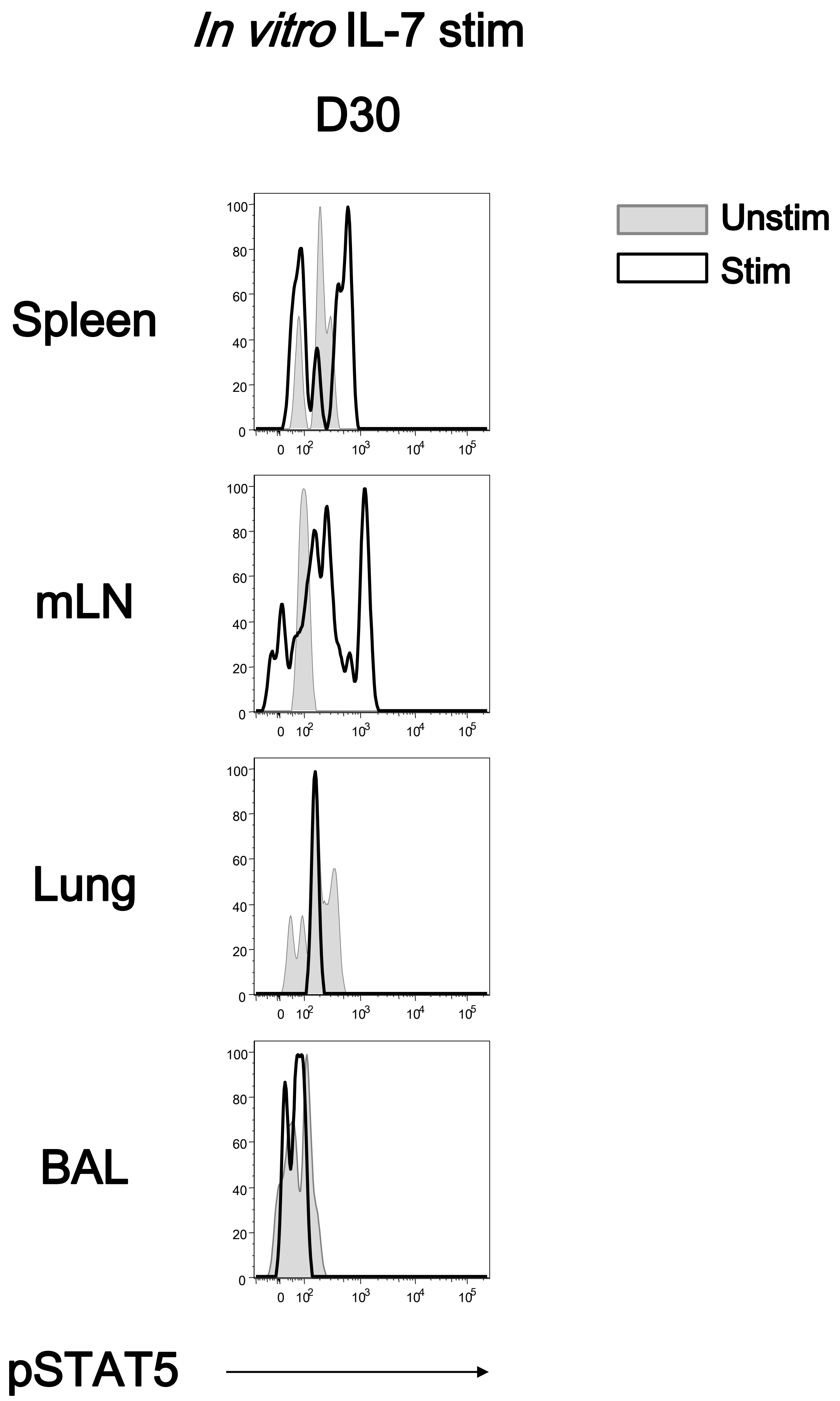

Supplementary Figure 1. Allergen-specific memory CD4 T cells responded to recall challenge. (a) Allergic asthma mice were rechallenged with a mixture of OVA and HDM once a day at day 22 and 23. (b) On day 25, the number of DO11.10 cells was counted. These data represent Mean ± SEM. (c) Isolated leukocytes from the airway and lung were prepared and stained with Diff-quik, and the number of eosinophils was counted. These data are representative of three independent experiments with two mice per group.

Supplementary Figure 2. Unique phenotypes of allergen-specific CD4 T cells in the lung and airways were observed. The expressions of (a) CD127, (b) CD27, and (c) CD62L of OVA-specific memory CD4 T cell were examined from the spleen, mLN, lung, and BAL on days 7, 30, and 70 post challenge using flow cytometry. These data are representative of nine independent experiments.

Supplementary Figure 3. Allergen-specific CD4 T cells in the lung and airways phosphorylated their STAT5 molecules upon IL-7 stimulation. Lymphocytes were prepared from the spleen, mLN, lung and airways, and then stimulated with IL-7 *in vitro* on day 30 post challenge. The levels of STAT5 phosphorylation in allergen-specific CD4 T cells from the spleen, mLN, lung and airways were examined using flow cytometry. These data are representative of three independent experiments.
